# Efficacy and safety of amrubicin monotherapy after atezolizumab plus carboplatin and etoposide in patients with relapsed small-cell lung cancer

**DOI:** 10.1007/s10637-022-01269-9

**Published:** 2022-06-24

**Authors:** Hisao Imai, Yoshiaki Nagai, Hiroyuki Minemura, Takeshi Tsuda, Yutaka Yamada, Satoshi Wasamoto, Takayuki Kishikawa, Ayako Shiono, Jun Shiihara, Ou Yamaguchi, Atsuto Mouri, Kyoichi Kaira, Kenya Kanazawa, Hirokazu Taniguchi, Koichi Minato, Hiroshi Kagamu

**Affiliations:** 1 Department of Respiratory Medicine, Comprehensive Cancer Center, International Medical Center, Saitama Medical University, 1397-1 Yamane, Hidaka, Saitama 350-1298 Japan; 2 Division of Respiratory Medicine, Gunma Prefectural Cancer Center, Ota, Gunma Japan; 3 Department of Respiratory Medicine, Jichi Medical University, Saitama Medical Center, Saitama, Saitama Japan; 4 Department of Pulmonary Medicine, Fukushima Medical University, Fukushima, Japan; 5 Division of Respiratory Medicine, Toyama Prefectural Central Hospital, Toyama, Toyama Japan; 6 Division of Respiratory Medicine, Ibaraki Prefectural Central Hospital, Kasama, Ibaraki Japan; 7 Division of Respiratory Medicine, Saku Central Hospital Advanced Care Center, Saku, Nagano Japan; 8 Division of Thoracic Oncology, Tochigi Cancer Center, Utsunomiya, Tochigi Japan

**Keywords:** Amrubicin, Atezolizumab plus carboplatin and etoposide, Immune checkpoint inhibitor, Relapsed small-cell lung cancer

## Abstract

This study examined the activity and safety of amrubicin monotherapy among relapsed small-cell lung cancer (SCLC) patients who had previously been treated with atezolizumab plus carboplatin and etoposide (AteCE). This retrospective study evaluated patients with relapsed SCLC who were treated with previously AteCE combination therapy followed by amrubicin monotherapy between August 2019 and May 2021. Clinical efficacy and toxicity were analyzed. Overall, 40 patients were included: 12 and 28 patients had sensitive and refractory relapse, respectively. The response rate was 32.5% (25.0% in the sensitive group and 35.7% in the refractory group). The median progression-free survival (PFS) and overall survival (OS) from the first amrubicin treatment was 3.4 months (95% CI: 1.9–4.9 months) and 9.9 months (95% CI: 4.5–11.5 months), respectively. There was no significant between-group difference in median PFS (3.6 months vs. 3.2 months, *p* = 0.42) or median OS (11.2 months vs. 7.3 months, *p* = 0.78). Grade ≥ 3 hematological adverse events occurred as follows: decreased white blood cells in 52.5% of patients; decreased neutrophil count in 57.5%; and febrile neutropenia in 10.0%. Grade 3 pneumonitis was observed in one patient. There were no treatment-related deaths. Amrubicin is feasible and effective for relapsed SCLC patients previously treated with AteCE therapy. Although immune checkpoint inhibitor treatment (ICI) does not improve the effect of amrubicin, the toxicity is not increased, suggesting that amrubicin remains effective even after ICI administration. Thus, amrubicin after AteCE could be the preferred standard chemotherapeutic choice in patients with relapsed SCLC.

**Supplementary Information:**

The online version contains supplementary material available at 10.1007/s10637-022-01269-9.

## Introduction

Lung cancer reportedly accounts for the largest number of cancer deaths globally [1]. Small-cell lung cancer (SCLC) comprises approximately 15% of all lung cancer cases and is an aggressive tumor characterized by prompt doubling time, high proliferation fraction, and early development of widespread metastases [2, 3]. Approximately two-thirds of SCLC cases have extensive disease (ED) at diagnosis, which correlates with poor prognosis [4]. Although most SCLC patients respond to initial treatment, long-term survival is poor. Unfortunately, disease progression or relapse is common [5–9]. Until recently, the standard first-line treatment for patients with ED-SCLC was combination chemotherapy of platinum and etoposide. However, the median overall survival (OS) is limited to approximately 10 months, and OS improvement has not improved in more than 20 years [10, 11]. Before the introduction of immune checkpoint inhibitors (ICIs), the reported outcomes of first-line chemotherapy in patients with ED-SCLC included a median progression-free survival (PFS) of 4.3–5.7 months, a median OS of 7.5–10.9 months, and an average 5-year survival rate of only 2.8% [11, 12].

Recently, ICIs have brought about survival benefits in patients with ED-SCLC [13–16]. Atezolizumab, which is a humanized monoclonal anti–programmed death ligand 1 (PD-L1) antibody, inhibits PD-L1–programmed death 1 (PD-1) and PD-L1–B7-1 signaling and restores tumor-specific T-cell immunity [17]. The landmark IMpower133 trial showed significantly better survival outcomes in atezolizumab plus carboplatin and etoposide (AteCE) therapy than in carboplatin and etoposide therapy [13, 14]. Moreover, durvalumab, another PD-L1 antibody, showed similar survival efficacy in the CASPIAN trial [15, 16].

Amrubicin and its active metabolite, amrubicinol, are inhibitors of DNA topoisomerase II and exert cytotoxicity by stabilizing cleavable complexes via topoisomerase II rather than via DNA intercalation. Amrubicinol is 5–100 times more active than amrubicin [18]. A previous phase III trial evaluating the activity of amrubicin in relapsed SCLC demonstrated that the overall response rate, PFS, and OS were 31.1%, 4.1 months, and 7.5 months, respectively [19]. Moreover, the response rate, PFS, and OS were 40.9%, 5.5 months, and 9.2 months, respectively, for sensitive cases, and 20.1%, 2.8 months, and 6.2 months, respectively, for refractory cases [19]. The most common adverse event (AE) associated with amrubicin administration is myelosuppression, such as leukopenia and neutropenia, with non-hematologic toxicities occurring less frequently [19–26].

Amrubicin monotherapy is an established standard second-line chemotherapeutic regimen in patients with refractory SCLC [26]. A meta-analysis reported that amrubicin monotherapy is effective in both sensitive and refractory cases of relapsed SCLC [27]. In patients with non-SCLC, overall response rates to cytotoxic single-agent chemotherapy after failure of anti-PD1 therapy are higher than responses to single-agent chemotherapy without prior anti-PD1 therapy [28]. There are also reports of improved treatment response of docetaxel plus ramucirumab therapy beyond nivolumab administration in pretreated non-SCLC [29], supporting the promising efficacy of cytotoxic anticancer drugs after ICIs.

Although systemic treatment consisting of ICIs plus combination chemotherapy with platinum and etoposide is the preferred therapeutic treatment for ED-SCLC, the effectiveness and toxicity of amrubicin in relapsed SCLC patients previously treated with AteCE have not been examined. Thus, the current study aimed to examine the activity and feasibility of amrubicin monotherapy among relapsed SCLC patients who have been pretreated with AteCE.

## Patients and methods

### Study design and patients

This retrospective study evaluated patients with relapsed SCLC who previously received AteCE combination treatment followed by amrubicin monotherapy between August 2019 and May 2021 in one of nine Japanese institutions. The eligibility criteria were: unresectable clinical stage (III or IV) disease or postoperative recurrence at first-line therapy, cytologically or histologically diagnosed SCLC, and first-line treatment with AteCE combination therapy followed by second-line amrubicin monotherapy.

All patients underwent systematic evaluation and standardized staging procedures before treatment. Clinical stage was assigned based on the results of physical examination, chest radiography, computed tomography (CT) scans of the chest and abdomen, CT or magnetic resonance imaging (MRI) of the brain, and bone scintigraphy or ^18^F-fluorodeoxyglucose positron emission tomography to assess the TNM stage. Pathological stage III/IV SCLC was determined based on the Union for International Cancer Control tumor-node-metastasis (TNM) Classification, 8th Edition. Sensitive relapse was defined as response to initial anticancer agent treatment and relapse within > 90 days beyond cytotoxic drug treatment. Meanwhile, refractory relapse was defined as no response to initial cytotoxic drug treatment or relapse within 90 days.

This study was approved by the Institutional Review Board of Saitama Medical University International Medical Center (No. 2021–113). All procedures complied with the ethical standards of the institutional and/or national research committee and with the 1964 Declaration of Helsinki and its subsequent amendments, or comparable ethical standards. Because this is a retrospective study, informed consent was waived.

### Treatment and response evaluation

All patients had no history of amrubicin monotherapy, and amrubicin was administered intravenously at dose of 25–45 mg/m^2^ on days 1 to 3 every 22 or 29 days. Granulocyte colony-stimulating factor was administered as prophylaxis for neutropenia at the discretion of the attending physician, but administration was not mandatory. Amrubicin monotherapy was continued until disease progression, occurrence of unacceptable toxicities, or patient refusal. Radiological tumor responses were assessed based on best overall treatment response and maximum tumor shrinkage according to the Response Evaluation Criteria in Solid Tumors, version 1.1 [30]. Patients who failed treatment were administered subsequent therapy if they wished, including continuation of amrubicin monotherapy. Treatment toxicities related to amrubicin administration were graded following the Common Terminology Criteria for Adverse Events (CTCAE version 5.0).

### Statistical analysis

Statistical analyses were performed using the Fisher’s exact test and the Welch’s t-test for categorical and continuous variables, respectively. PFS was calculated from the first day of treatment until progressive disease or any-cause death. OS was calculated from the first day of treatment until death or was censored on the date of the last follow-up. The Kaplan–Meier method was used to estimate survival as a function of time, and survival differences were analyzed using log-rank tests. For the univariable and multivariable prognostic assessments of several clinical important parameters, the Cox proportional hazards regression model was used to calculate hazard ratios (HR) and 95% confidence intervals (CI). For evaluation of correlation, we used the Spearman rank correlation analysis and linear regression analysis. Differences were considered significant at a two-tailed *p*-value of < 0.05. All statistical analyses were performed using JMP, version 11.0, for Windows (SAS Institute, Cary, NC, USA).

## Results

### Patient characteristics

Forty patients were treated with AteCE combination chemotherapy followed by amrubicin as second-line chemotherapy. Table 1 shows the patient characteristics. Twelve patients with sensitive relapse and 28 patients with refractory relapse were evaluable for treatment response, survival, and safety. The patients were predominantly male (32, 80.0%), and the median age at the initiation of amrubicin administration was 71 years (range, 57–84 years). Performance status (PS) at the time of amrubicin initiation was 0–1 in 35 patients (87.5%). The median number of prior AteCE cycles was 4 (range, 4–5) in the sensitive group and 4 (range, 2–6) in the refractory group. No patient in the sensitive group and six patients in the refractory group received additional atezolizumab during the course of carboplatin and etoposide combination chemotherapy.

**Table 1:** Baseline patient characteristics (n = 40)

Characteristic	Sensitive group	Refractory group	Total
Number of patients	12	28	40
Sex			
Male	10	22	32
Female	2	6	8
Age at the start of AMR, (years)			
Median	71.5	71	71
Range	57–84	58–82	57–84
Smoking			
Yes	11	27	38
No	1	1	2
ECOG-PS at the start of AMR			
0	2	7	9
1	9	17	26
2	0	4	4
≥ 3	1	0	1
Histology			
Small cell carcinoma	12	27	39
Combined small cell carcinoma	0	1	1
Disease extent			
IV	12	27	39
Postoperative recurrence	0	1	1
History of postoperative adjuvant chemotherapy			
Yes	0	1	1
No	12	27	39
Prior therapy			
Atezolizumab plus carboplatin and etoposide alone	12	27	39
Surgery plus adjuvant chemotherapy followed by atezolizumab plus carboplatin and etoposide	0	1	1
Intracranial metastases at initial treatment			
Yes	3	11	14
No	9	17	26
Liver metastases at initial treatment			
Yes	4	5	9
No	8	23	31
Bone metastases at initial treatment			
Yes	5	12	17
No	7	16	23
Number of cycles prior chemotherapy administered			
Median	4	4	4
Range	4–5	2–6	2–6
Addition of atezolizumab in the course of carboplatin and etoposide			
Yes	0	6	6
No	12	22	34
Number of cycles of atezolizumab maintenance therapy administered			
Median	2.5	2	2
Range	0–12	0–8	0–12
Reason for discontinuation of AteCE administration^a^			
Progressive disease	11	26	37
Adverse events	1	1	2
Others	0	1	1
Response to prior treatment			
CR	3	0	3
PR	7	21	28
SD	0	6	6
PD	2	1	3
NE	0	0	0
Continuing administration of AMR at data cutoff	1	1	2

*AMR* amrubicin, *ECOG-PS* Eastern Cooperative Oncology Group-Performance status, *AteCE* atezolizumab plus carboplatin and etoposide, *CR* complete response, *PR* partial response, *SD* stable disease, *PD* progressive disease, *NE* not evaluated

^a^Including atezolizumab maintenance therapy

#### Treatment response and delivery

Treatment response and therapeutic delivery according to the patient group are shown in Table 2. The overall response and disease control rates in the entire cohort were 32.5% (95% CI: 20.0–48.0) and 60.0% (95% CI: 44.5–73.6), respectively. The response rate was 25.0% (95% CI: 8.2–53.8) in the sensitive group and 35.7% (95% CI: 20.6–54.2) in the refractory group. These differences were not significant.

**Table 2:** Tumor response to therapy and treatment delivery

Characteristic	Total	Sensitive group	Refractory group	*p*-value^a^
Response				
CR	1	1	0	
PR	12	2	10	
SD	11	2	9	
PD	14	5	9	
NE	2	2	0	
Response rate (%) (95% CI)	32.5 (20.0–48.0)	25.0 (8.2–53.8)	35.7 (20.6–54.2)	0.71
Disease control rate (%) (95% CI)	60.0 (44.5–73.6)	41.6 (19.2–68.1)	67.8 (49.2–82.1)	0.16
No. of treatment cycles				
Median	3	3	3.5	0.85^b^
Range	1–14	1–14	1–13	
Dose (mg/m^2^ per day)				
25	1	0	1	
30	10	1	9	
35	17	7	10	
40	11	4	7	
45	1	0	1	
Dose reduction				
Starting dose 25–35 mg/m^2^ per day				
Yes/no	3/25	1/7	2/18	> 0.99
Starting dose 40–45 mg/m^2^ per day				
Yes/no	3/9	0/4	3/5	0.49

*CR* complete response, *PR* partial response, *SD* stable disease, *PD* progressive disease, *NE* not evaluate, *95% CI* 95% confidence interval

^a^Comparison between sensitive group and refractory group

^b^Welch’s t-test

The median number of administration cycles was 3 (range, 1–14) in the sensitive group and 3.5 (range, 1–13) in the refractory group. The most common starting dose was 35 mg/m^2^/day for both groups. Dose reduction of amrubicin was more frequent at a starting dose of ≥ 40 mg/m^2^/day than at ≤ 35 mg/m^2^/day (33.3% [3/9] vs. 12.0% [3/25]).

#### Survival

**Fig. 1:**
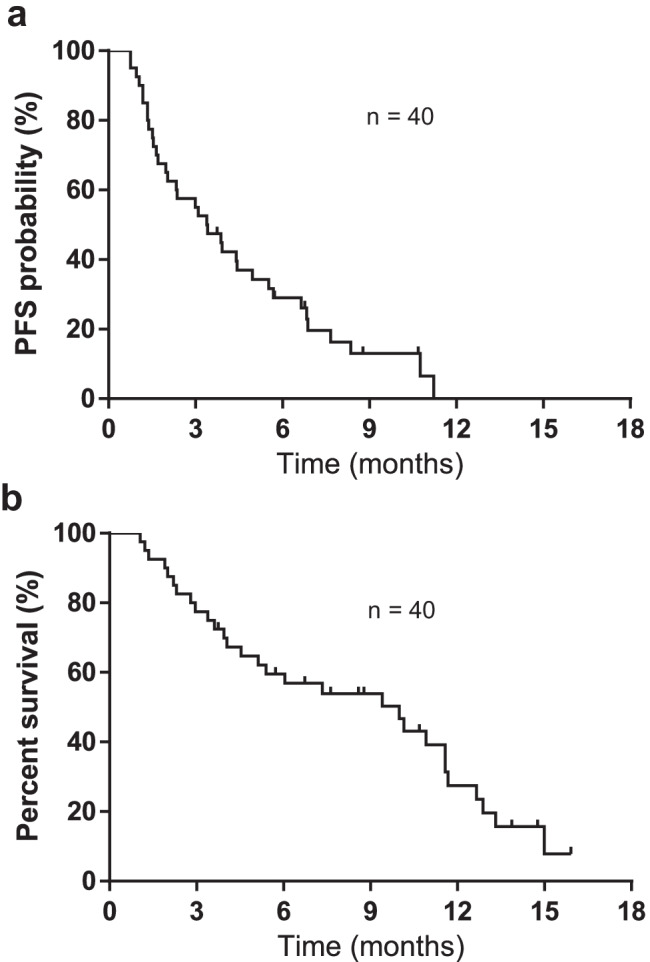
Kaplan–Meier analysis of survival in the 40 patients. **a** Progression-free survival (PFS) (median: 3.4 months). **b** Overall survival (OS) (median: 9.9 months)

**Fig. 2:**
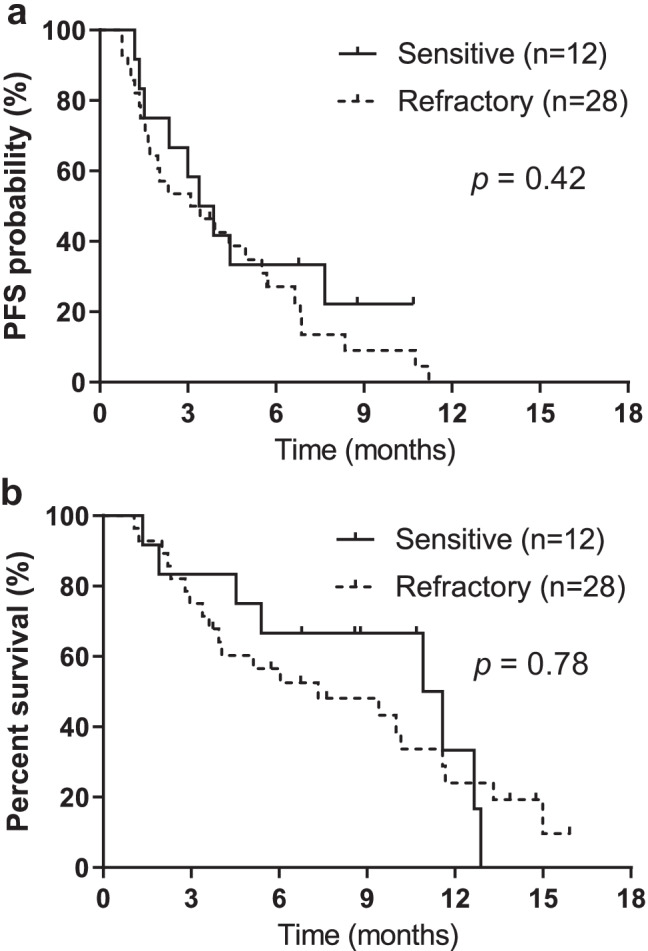
Survival by relapse group. **a** The median progression-free survival (PFS) in the sensitive- and refractory- relapse groups is 3.6 and 3.2 months, respectively (*p* = 0.42). **b** The median overall survival (OS) in the sensitive and refractory relapse groups is 11.2 and 7.3 months, respectively (*p* = 0.78)

The median follow-up period in the overall population was 6.8 months (range, 1.1–15.9 months), and the median PFS and OS from the initial amrubicin monotherapy for all 40 patients was 3.4 (95% CI: 1.9–4.9) and 9.9 months (95% CI: 4.5–11.5), respectively (Fig. 1a, b). PFS and OS based on relapse pattern is demonstrated in Fig. 2a and b, respectively. The median PFS was 3.6 months in the sensitive group and 3.2 months in the refractory group (*p* = 0.42). The median OS was 11.2 months in the sensitive group and 7.3 months in the refractory group (*p* = 0.78). In the univariate analysis, PS was an influencing factor of PFS, and the multivariate analysis showed that PS at the time of amrubicin administration was an independent prognostic factor for PFS. Meanwhile, the number of cycles of atezolizumab maintenance therapy was a prognostic factor for PFS only in the univariate analysis. For OS, univariate and multivariate analyses demonstrated that a PS of 0–1 or ≥ 2 at the initiation of amrubicin administration was an independent prognostic factor for OS (Table 3).

**Table 3:** Univariate and multivariate analyses for progression-free survival and overall survival

		Univariate analysis			Multivariate analysis				Univariate analysis			Multivariate analysis		
		PFS			PFS				OS			OS		
Factors	Median PFS (months)	HR	95% CI	*p*-value	HR	95% CI	*p*-value	Median OS (months)	HR	95% CI	*p*-value	HR	95% CI	*p*-value
Sex														
Male/female	3.8/2.7	0.97	0.42–2.61	0.95				9.9/8.3	0.75	0.30–2.27	0.58			
ECOG-PS														
0–1/ ≥ 2	3.9/1.2	0.11	0.04–0.36	**0.0005**	0.15	0.05–0.47	**0.0021**	10.9/1.7	0.06	0.01–0.22	**< 0.0001**	0.06	0.01–0.24	**< 0.0001**
Age (years)														
< 70/ ≥ 70	2.9/3.8	0.96	0.46–1.92	0.92				10.1/9.4	1.32	0.61–2.78	0.46			
Relapse pattern														
Sensitive/refractory	3.6/3.2	0.73	0.32–1.52	0.42				11.2/7.3	0.88	0.36–1.97	0.77			
Dose (mg/m^2^ per day)														
25–35/40–45	2.7/6.6	1.87	0.87–4.45	0.10				9.9/9.4	1.14	0.50–2.94	0.75			
Number of cycles of atezolizumab maintenance therapy														
< 2/ ≥ 2	1.7/4.4	2.47	1.18–5.01	**0.01**	2.01	0.92–4.21	0.07	5.3/9.9	1.53	0.67–3.29	0.29	1.05	0.42–2.41	0.91
Efficacy of AteCE														
PR/non-PR	3.9/1.6	0.47	0.22–1.15	0.09				9.4/10.9	0.84	0.36–2.29	0.71			
Presence of irAE in previous treatment														
Yes/no	3.9/3.0	0.93	0.27–2.43	0.90				10.1/9.4	0.81	0.19–2.33	0.72			

Bold-type *p* values are statistically significant (*p* < 0.05)

*PFS* progression-free survival, *OS* overall survival, *HR* hazard ratio, *95% CI* 95% confidence interval, *ECOG-PS* Eastern Cooperative Oncology Group-Performance status, *AteCE* atezolizumab plus carboplatin and etoposide, *PR* partial response, *irAE* immune-related adverse event

**Fig. 3:**
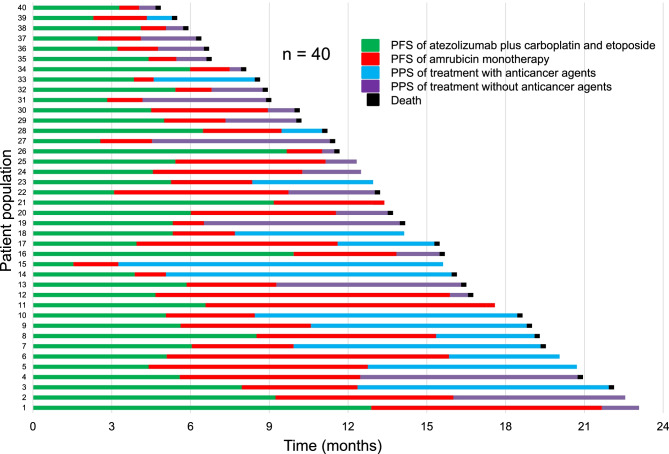
Progression-free survival (PFS) of atezolizumab plus carboplatin and etoposide (AteCE), PFS of amrubicin monotherapy, and post-progression survival (PPS) in the overall cohort (n = 40)

**Fig. 4:**
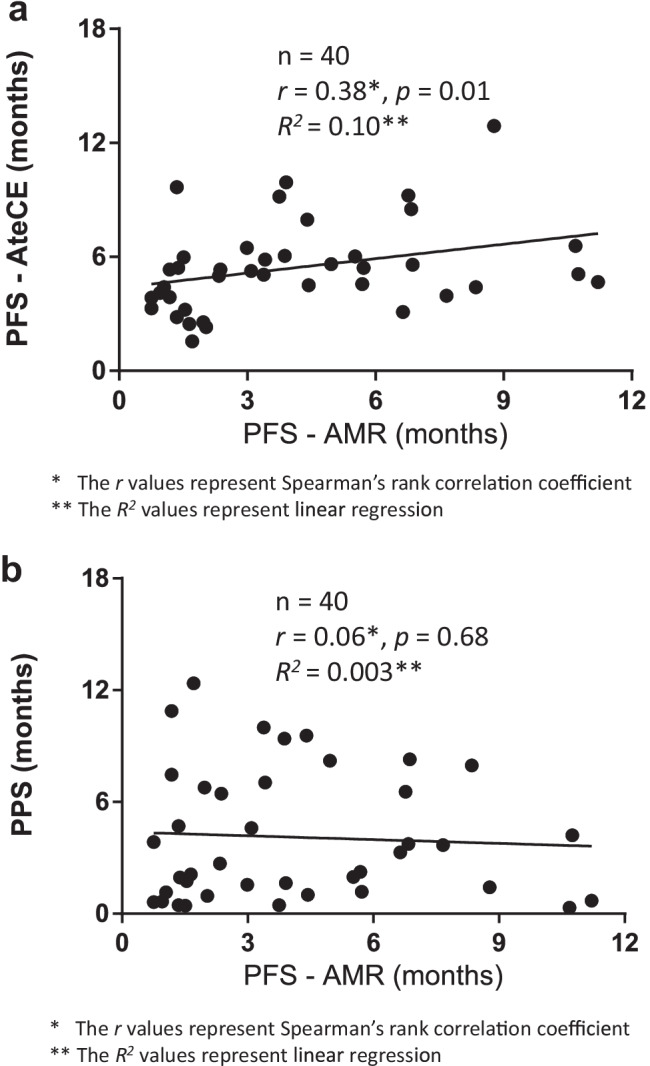
Correlation of progression-free survival (PFS) with amrubicin (AMR). **a** Correlation of PFS with atezolizumab plus carboplatin and etoposide (AteCE). **b** Correlation of post-progression survival with AMR

Figure 3 shows the PFS of AteCE, PFS of amrubicin monotherapy, and post-progression survival (PPS) of amrubicin monotherapy in the entire cohort. The relationship of PFS of amrubicin monotherapy with PFS of AteCE therapy and with PPS of amrubicin monotherapy is demonstrated in Fig. 4a and b. Spearman’s rank correlation coefficient and linear regression revealed that the PFS of AteCE therapy was weakly associated with that of amrubicin monotherapy (*r* = 0.38, *p* = 0.01, *R*^*2*^ = 0.10), whereas the PPS of amrubicin monotherapy was not associated with that of amrubicin monotherapy (*r* = 0.06, *p* = 0.68, *R*^*2*^ = 0.003).

The median PFS of AteCE was 5.1 months (95% CI: 4.4–5.5) for the entire cohort (Online Resource 1). The median OS from the first AteCE combination chemotherapy for the entire cohort was 15.4 months (95% CI: 11.3–18.4) (Online Resource 2).

#### Toxicity

Treatment-related AEs are shown in Table 4. All 40 patients were assessed for drug-related AEs. The most common treatment-related AE was myelosuppression with 52.5% of patients developing a grade 3–4 decrease in white blood cells, and 57.5% of patients developing a grade 3–4 decrease in neutrophil count. Febrile neutropenia occurred in four patients (10.0%). Grade 3–4 anemia occurred in one patient (2.5%), and a grade 3–4 decrease in platelet count was observed in five patients (12.5%). The rate of non-hematologic AEs was low. The occurrence of immune-related AEs was also low. The most common grade 3–4 non-hematologic AE was infection (7.5%). Grade 3 pneumonitis occurred in one patient. No cardiotoxicity and treatment-related death was observed.

**Table 4:** Patients with amrubicin treatment-related adverse events (CTCAE v5.0)

Event	Grade 3	Grade 4	Grade 5	≥ Grade 3	%
White blood cell decreased					
Total	13	8	-	21	52.5
Dose of 25–35 mg/m^2^/day	7	6	-	13	46.4
Dose of 40–45 mg/m^2^/day	6	2	-	8	66.6
Neutrophil count decreased					
Total	9	14	-	23	57.5
Dose of 25–35 mg/m^2^/day	6	9	-	15	53.5
Dose of 40–45 mg/m^2^/day	3	5	-	8	66.6
Anemia					
Total	1	0	0	1	2.5
Dose of 25–35 mg/m^2^/day	1	0	0	1	3.5
Dose of 40–45 mg/m^2^/day	0	0	0	0	0
Platelet count decreased					
Total	3	2	-	5	12.5
Dose of 25–35 mg/m^2^/day	3	1	-	4	14.2
Dose of 40–45 mg/m^2^/day	0	1	-	1	8.3
Febrile neutropenia					
Total	4	0	0	4	10
Dose of 25–35 mg/m^2^/day	3	0	0	3	10.7
Dose of 40–45 mg/m^2^/day	1	0	0	1	8.3
Malaise	1	-	-	1	2.5
Pneumothorax	0	1	0	1	2.5
Infection	3	0	0	3	7.5
Pneumonitis	1	0	0	1	2.5

*CTCAE* Common Terminology Criteria for Adverse Events

Next, we analyzed hematologic AEs according to administration dose. The grade 3–4 hematologic AEs are shown in Table 4. Compared with patients receiving ≥ 40 mg/m^2^/day, patients receiving ≤ 35 mg/m^2^/day showed lower frequencies of decreased neutrophil count (46.4% vs. 66.6%) and decreased white blood cell count (46.4% vs. 66.6%), and higher frequencies of anemia (3.5% vs. 0%) and decreased platelet count (14.2% vs. 8.3%). Febrile neutropenia developed in 10.7% and 8.3% of patients receiving ≤ 35 mg/m^2^/day and ≥ 40 mg/m^2^/day, respectively. Hematologic AEs developing at a dose of ≤ 35 mg/m^2^/day were not necessarily less frequent than those at ≥ 40 mg/m^2^/day.

#### Subsequent treatments

Treatments administered following amrubicin monotherapy are shown in Online Resource 3. Among the 35 patients who developed disease progression, 15 patients received anticancer treatments. For subsequent therapy following progressive disease, the most common third-line treatment was topotecan monotherapy (n = 10; 66.6%) followed by irinotecan monotherapy (n = 3; 20.0%). Twenty patients received best supportive care alone. Five patients remained on amrubicin monotherapy or were off amrubicin monotherapy, but showed no evidence of disease progression.

## Discussion

In this study, second-line amrubicin monotherapy after AteCE combination chemotherapy showed favorable effectiveness and no new safety concerns. Therapeutic choices for patients with relapsed SCLC remain limited and unclear. Amrubicin is often the treatment of choice for patients with refractory or relapsed SCLC, but the clinical effectiveness and feasibility of amrubicin in patients with relapsed SCLC treated with AteCE have not been assessed. To our best knowledge, this is the first analysis of the treatment effectiveness and feasibility of amrubicin monotherapy for relapsed SCLC patients treated with AteCE therapy.

Patients with SCLC are commonly resistant to cytotoxic drug chemotherapy beyond the first-line setting, but subsequent treatment options are scarce [31]. This study demonstrated that amrubicin monotherapy after AteCE is effective for relapsed SCLC. Amrubicin and topotecan are anticancer drugs that have shown clinical benefit in the second-line setting [19, 21]. Amrubicin has been evaluated in several studies (Table 5); however, there are no reports of amrubicin monotherapy after ICIs. The efficacy of the second-line treatment usually relies on the responsiveness of the tumor to the first-line chemotherapy; that is, whether the tumor is sensitive or refractory.

**Table 5:** Reports of the amrubicin monotherapy for relapsed small-cell lung cancer

Author (year)	Study design	No. of patients	Dosage (mg/m^2^)	Median age	PS 0/1/2/3/4	Prior ICI therapy	Relapse cutoff period	Sensitive/refractory	ORR (%) Total (Sensitive/refractory)	PFS (months) Total (Sensitive/refractory)	OS (months) Total (Sensitive/refractory)
Onoda (2006)	2	60	40	67	28/28/4/0/0	No	60	44/16	52 (52/50)	NR (4.2/2.6)	11.2 (11.6/10.6)
Inoue (2008)	r2	29^a^	40	70	14/10/5/0/0	No	90	17/12	38 (53/17)	3.5 (3.9/2.6)	8.1 (9.9/5.3)
Shimokawa (2009)	R	32	25–45	66	0–2/3–4: 28/4	No	60	17/15	53.1 (70.6/33.3)	3.2 (NR/NR)	5.5 (5.5/5.5)
Kim (2010)	R	69	25–45	66	0–1/2–4: 48/28	No	90	27/42	51 (70/38)	NR (3.2/1.9)	NR (6.2/4.8)
Kaira (2010)^b^	2	29	35	67	12/12/5/0/0	No	90	10/19	44.8 (60/36.8)	4.0 (4.0/4.0)	12.0 (12.0/11.0)
Ettinger (2010)	2	75	40	63	24/38/13/0/0	No	90	0/75	21.3 (–/21.3)	3.2 (–/3.2)	6.0 (–/6.0)
Jotte (2011)	r2	50^a^	40	63	20/24/6/0/0	No	90	50/0	44 (44/–)	4.5 (4.5/–)	9.2 (9.2/–)
Murakami (2014)	2	82	40	66	34/48/0/0/0	No	90	0/82	32.9 (–/32.9)	3.5 (–/3.5)	8.9 (–/8.9)
von Pawel (2014)	3	424^a^	40	62	126/289/9/0/0	No	90	225/199	31.1 (40.9/20.1)	4.1 (5.5/2.8)	7.5 (9.2/6.2)
Current study	R	40	25–45	71	9/26/4/1/0	Durvalmab	90	12/28	32.5 (25.0/35.7)	3.4 (3.6/3.2)	9.9 (11.2/7.3)

*NSCLC* non-small-cell lung cancer, *SCLC* small-cell lung cancer, *N* number, *PS* performance status, *ICI* immune checkpoint inhibitor, *ORR* overall response rate, *PFS* progression-free survival, *OS* overall survival, *R* retrospective, *NR* not reported

^a^Amrubicin arm

^b^The report assesses both NSCLC and SCLC. Only data assessing SCLC is referenced

In previous studies, the overall response rate was higher in sensitive cases (40.9%–70.6%) than in refractory cases (17%–50%). The PFS for sensitive and refractory cases was 3.2–5.5 months and 1.9–4.0 months, respectively, and the OS was 5.5–12.0 months and 4.8–11.0, respectively [19–26, 32]. In the current study, the response rate was 32.5% for all cases, 25.0% for sensitive cases, and 35.7% for refractory cases. Although the current analysis was a retrospective study, the response rate in sensitive cases was inferior to that in other reports. However, the response rate in refractory cases was similar or better than that in other reports, and the response rate was reasonable.

The low response rate in the sensitive group might be attributed to the rather small number of cases (n = 12), differences in patient characteristics, or other biases. However, the PFS and OS in this analysis was not significantly different between sensitive and refractory cases. As shown in Table 5, the PFS and OS were comparable or better in sensitive and refractory cases than in other prospective and retrospective studies reported to date. These results indicate that amrubicin monotherapy after AteCE can be a helpful chemotherapeutic option for chemotherapy-resistant/refractory patients.

In the multivariate analysis of PFS and OS, PS at the beginning of second-line amrubicin monotherapy was an independent prognostic factor for PFS and OS. Although the number of cycles of atezolizumab maintenance therapy (< 2/ ≥ 2) was also found to be correlated with PFS in the univariate analysis, it was not an independent prognostic factor in the multivariate analysis. These findings indicate that amrubicin administration might be effective for improving PFS and OS in refractory or relapsed SCLC patients with favorable PS. The Eastern Cooperative Oncology Group-Performance Status, a subjective scoring system that assesses the general condition of cancer patients, has been reported to be a strong prognostic predictor, showing independent correlations with PFS and OS [33].

The current analysis confirms that PS is a strong prognostic factor, as reported in previous investigations [33], suggesting that our study population reflects the general patient population. The present analysis did not identify the commonly reported relapse pattern (sensitive/refractory) as an independent prognostic factor for either PFS or OS. However, the median PFS and OS of the sensitive group were longer than those of the refractory group, although the difference was not significant (Fig. 2). This result may be due to the small number of cases in both groups. Moreover, prior to the era of ICIs in SCLC, patients who respond to initial pharmacotherapy and have a long interval between completion of initial therapy and relapse (usually ≥ 60–90 days) are often defined as having “sensitive relapse,” while others are defined as “refractory relapse.” Sensitive relapse patients respond better to pharmacotherapy at relapse and survive longer [34, 35]. However, it may be necessary to re-evaluate the criteria for sensitive or refractory relapse after the use of ICIs.

A randomized phase III trial (GFPC01-13) compared oral topotecan monotherapy with carboplatin and etoposide (platinum re-administration) in patients with sensitive relapse after platinum and etoposide therapy. The primary endpoint of PFS was significantly longer in the carboplatin and etoposide group (median: 4.7 months vs. 2.7 months, HR: 0.57, 90% CI: 0.41–0.73, *p* = 0.0041) [36]. In the current analysis, it might be possible that platinum re-administration may have been the treatment of choice in sensitive relapse cases in the participating institutions, resulting in a relative paucity of amrubicin monotherapy. In addition, univariate analysis demonstrated that PFS was better in the group with more frequent atezolizumab maintenance therapy (≥ 2).

Although multivariate analysis showed no significant difference in the number of cycles of atezolizumab maintenance treatment, there was a weak correlation between the PFS of AteCE and that of amrubicin monotherapy (Fig. 4a). The results suggest that a longer PFS with AteCE also results in a longer PFS for amrubicin monotherapy. Preclinical data suggests that anthracyclines (e.g., amrubicin) as anticancer agents can induce immunogenic cell death in sensitive human tumor cells [37]. However, there are no clinical data reporting the efficacy of anthracyclines (e.g., amrubicin) after ICIs.

As shown in Online Resource 2, the OS from initiation of AteCE was 15.4 months, which compares favorably with the OS in previous phase III trials [13, 14]. A previous report described that PPS has a greater effect on OS than om PFS in refractory SCLC patients who have received second-line amrubicin monotherapy [38]. As shown in Fig. 3 and Online Resource 1, 15 patients received amrubicin monotherapy followed by anticancer drug therapy and 20 patients received best supportive care alone. Topotecan was the most common treatment administered after third-line treatment, followed by irinotecan. Given that etoposide was used for first-line treatment in all patients, it will be necessary to examine the impact of these cytotoxic drug agents on anticancer drug treatment after AteCE combination therapy.

Results of a single-arm phase II trial of pembrolizumab, an ICI, in combination with amrubicin in patients with relapsed SCLC were recently reported [39]. Patients had not received any prior ICIs, but the response rate was 52.0%, the median PFS was 4.0 months, and the median OS was 10.6 months. Additionally, the common grade ≥ 3 AEs were neutropenia (64%), leukopenia (40%), and febrile neutropenia (16%). This report was focused on a regimen of pembrolizumab combined with amrubicin at the time of relapse, and the results showed it was effective and well-tolerated. In the future, it will be necessary to consider whether the treatment strategy for SCLC should be platinum combination chemotherapy with ICIs followed by cytotoxic anticancer agents or platinum combination chemotherapy followed by ICIs and cytotoxic anticancer agents such as amrubicin.

The adverse event profile of amrubicin monotherapy after AteCE noted in the current investigation indicates the feasibility of this modality, similar to previous phase II and III studies in which myelosuppression was observed as the common AE [19–21, 24–26]. Furthermore, there were no immune-related AEs which are characteristic of amrubicin toxicity after ICI use. The hematologic toxicities that developed were manageable AEs. Overall, non-hematologic AEs were mild. Furthermore, no new adverse event symptoms were observed with amrubicin monotherapy, even after AteCE combination therapy, and no immune-related AEs occurred. The frequency of grade ≥ 3 white blood cell count decrease and neutrophil count decrease was higher at a dose of ≥ 40 mg/m^2^/day than at a dose of ≤ 35 mg/m^2^/day. Moreover, dose reduction was more common at a dose of 40 mg/m^2^/day (Table 2).

As shown in the univariate and multivariate analyses, the initial dose (25–35 or 40–45 mg/m^2^/day) was not an independent prognostic factor influencing PFS and OS (Table 3). A previous report suggested that among patients with relapsed SCLC, those treated with amrubicin 35 mg/m^2^ attain comparable clinical benefit, but with less toxicity, than those treated with amrubicin 40 mg/m^2^ [32]. Therefore, in patients with relapsed SCLC, treatment with amrubicin ≤ 35 mg/m^2^/day may have comparable effects with less toxicity than treatment with ≥ 40 mg/m^2^/day or more. Therefore, the optimal dose of amrubicin monotherapy after AteCE combination therapy cannot be determined at this time, but it may not necessarily be > 40 mg/m^2^/day. This is an issue for further investigation. Pneumonitis was observed in one patient, but this patient recovered with steroid administration. No treatment-related death occurred. These findings indicate that with respect to toxicity, amrubicin monotherapy after AteCE is feasible for relapsed SCLC.

This study had some limitations. First, this was a retrospective study with a small sample size, and larger prospective studies are needed to validate our findings. Second, treatment with anticancer agents was reduced, skipped, or delayed at the discretion of the treating physician. However, we included all consecutive patients treated at the study sites to reduce this bias to the greatest extent possible, and the clinical charts were thoroughly reviewed. Third, the use of AteCE combination chemotherapy for first-line chemotherapy and amrubicin monotherapy for second-line chemotherapy was decided by the treating physician. These decisions could have introduced selection bias, which is an inherent limitation of retrospective studies. The possibility that this may have affected survival could not be ruled out.

In conclusion, amrubicin might be an effective and feasible treatment choice for patients with relapsed SCLC treated with AteCE therapy. Although ICI administration does not improve the effect of amrubicin, it did not enhance toxicity. This indicates that amrubicin is still effective in patients with relapsed SCLC, even after ICI administration. These findings may provide a new direction in the drug treatment of patients with refractory or relapsed SCLC.

## Supplementary Information

Below is the link to the electronic supplementary material.

Supplementary file1 (PPTX 68 KB)

Supplementary file2 (PPTX 70 KB)

Supplementary file3 (DOCX 25 KB)

## Data Availability

The data that support the findings of this study are available on request from the corresponding author. The data are not publicly available due to privacy or ethical restrictions.
