# Supplementary figures and images for: Efficacy and safety of amrubicin monotherapy after atezolizumab plus carboplatin and etoposide in patients with relapsed small-cell lung cancer
Source: Invest New Drugs. 2022 Jun 24;40(5):1066–79. doi: 10.1007/s10637-022-01269-9 (PMC9395483; doi:10.1007/s10637-022-01269-9)

## Slide 1
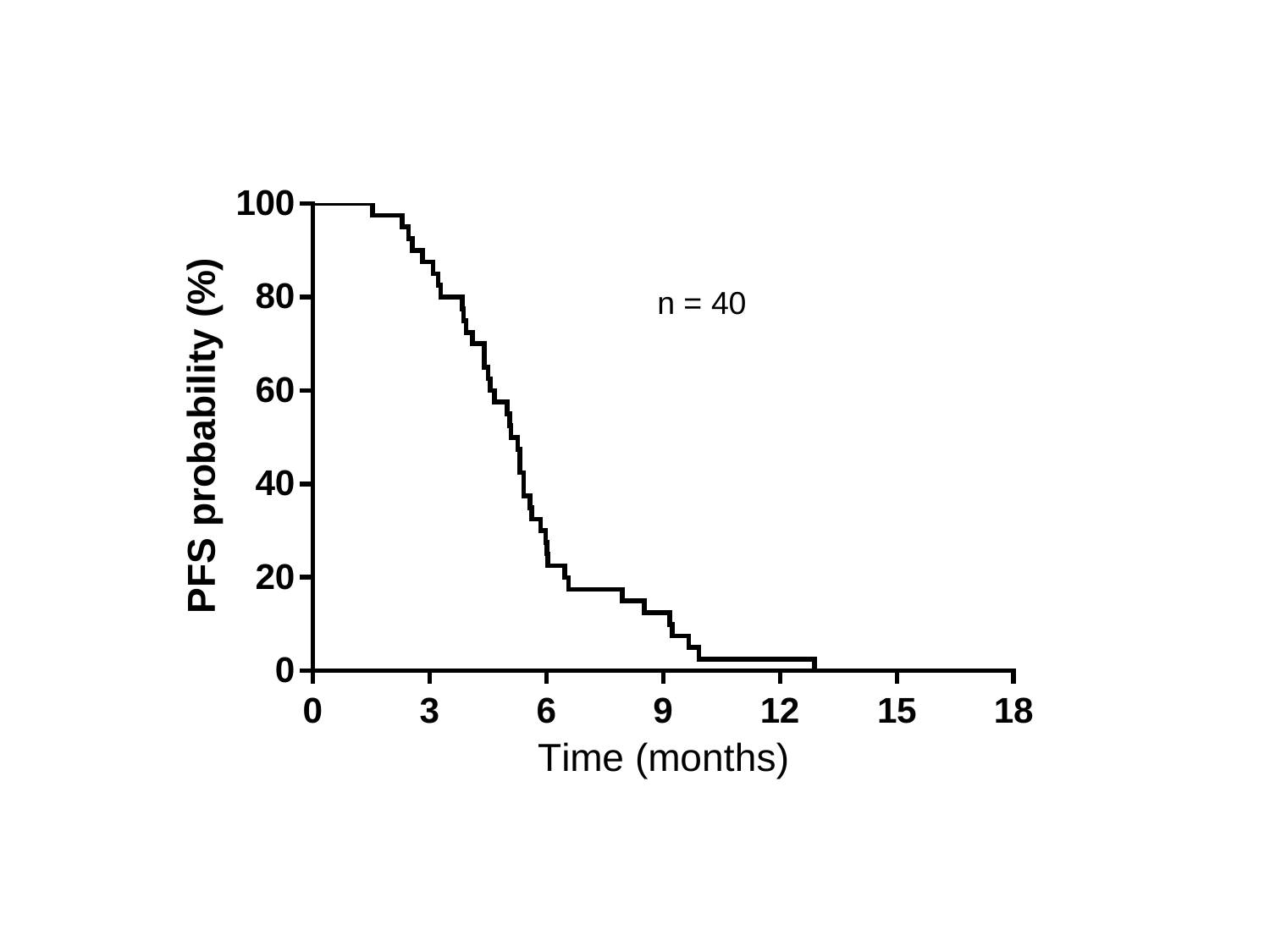

n = 40

Supplement: Supplementary file 1 — Supplementary file1 (PPTX 68 KB) [file 10637_2022_1269_MOESM1_ESM.pptx]

## Slide 1
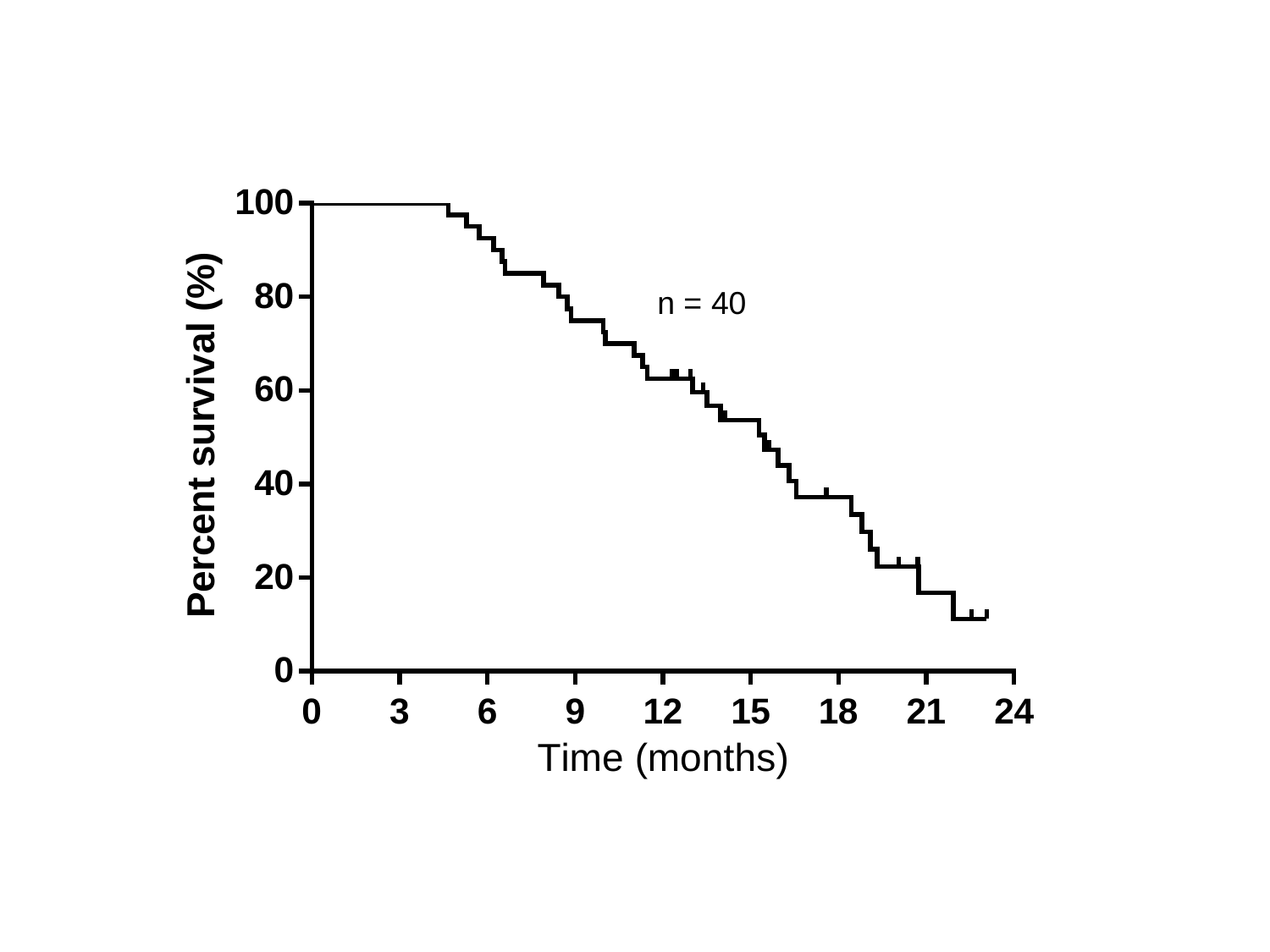

n = 40

Supplement: Supplementary file 2 — Supplementary file2 (PPTX 70 KB) [file 10637_2022_1269_MOESM2_ESM.pptx]
