# Supplementary material for: Efficacy and safety of amrubicin monotherapy after atezolizumab plus carboplatin and etoposide in patients with relapsed small-cell lung cancer
Source: Invest New Drugs. 2022 Jun 24;40(5):1066–79. doi: 10.1007/s10637-022-01269-9 (PMC9395483; doi:10.1007/s10637-022-01269-9)
Supplement: Supplementary file 3 — Supplementary file3 (DOCX 25 KB) [file 10637_2022_1269_MOESM3_ESM.docx]

Article title: Efficacy and safety of amrubicin monotherapy after atezolizumab plus carboplatin and etoposide in patients with relapsed small-cell lung cancer

Journal: Investigational New Drugs

Author Names: Imai et al.

Affiliation: Department of Respiratory Medicine, Comprehensive Cancer Center, International Medical Center, Saitama Medical University

Corresponding author: Hisao Imai, E-mail: [m06701014@gunma-u.ac.jp](mailto:m06701014@gunma-u.ac.jp)

Online Resource 3. Chemotherapy regimens administered following disease progression after second-line amrubicin monotherapy

|  | Third-line | Fourth-line | More than fifth-line | Total |
| --- | --- | --- | --- | --- |
| Topotecan | 10 | 1 | 0 | 11 |
| Irinotecan | 3 | 2 | 1 | 6 |
| Carboplatin+paclitaxel | 1 | 1 | 1 | 3 |
| Carboplatin+etoposide rechallenge | 1 | 1 | 1 | 3 |
| Amrubicin rechallenge | 0 | 1 | 0 | 1 |
| Immune checkpoint inhibitor | 0 | 1 | 0 | 1 |
| Others | 0 | 0 | 1 | 1 |
| Best supportive care | 20 | - | - | 20 |
